# Phylogenomic Analysis of Salmonella enterica Serovar Indiana ST17, an Emerging Multidrug-Resistant Clone in China

**DOI:** 10.1128/spectrum.00115-22

**Published:** 2022-07-05

**Authors:** Zengfeng Zhang, Jiang Chang, Xuebin Xu, Mengjun Hu, Shoukui He, Xiaojie Qin, Min Zhou, Chunlei Shi, Xianming Shi

**Affiliations:** a Department of Food Science & Technology, School of Agriculture & Biology, and State Key Lab of Microbial Metabolism, Shanghai Jiao Tong University, Shanghai, China; b Department of Microbiology, Shanghai Municipal Center for Disease Control and Prevention, Shanghai, China; c School of Food Science and Engineering, Wuhan Polytechnic University, Wuhan, China; University of Maryland Eastern Shore

**Keywords:** *Salmonella* Indiana, ST17, ciprofloxacin and ceftriaxone resistance, plasmids

## Abstract

Salmonella enterica serovar Indiana (*S.* Indiana) is an extremely expanded foodborne pathogen in China in recent years. This study aimed to elucidate the national prevalence and phylogenomic characterization of this pathogen in China. Among 5, 287 serotyped Salmonella isolates collected during 2002 to 2018, 466 *S.* Indiana isolates were found in 15 provinces, and 407 were identified to be ST17, and the rest were ST2040. Among 407 ST17 isolates, 372 (91.4%) were multidrug resistant, and 366 (89.9%) were resistant to ciprofloxacin, 235 (57.7%) were further resistant to ceftriaxone. Phylogenomic analysis revealed that ST17 isolates were classified into four clades (I, II, III and IV), which appeared in international clonal dissemination. ST17 isolates from China fell into Clade IV with part of isolates from the United Kingdom, the United States, South Korea, and Thailand, suggesting their close genetic relationship. Mutations in quinolone resistance-determining regions (QRDR) of GyrA and ParC, and plasmid-mediated quinolone resistance (PMQR) genes *aac*(6′)-Ib-cr, *oqx*AB, and *qnr*S as well as extended spectrum β-lactamases (ESBL) genes *bla*_CTX-M_, *bla*_OXA_, and *bla*_TEM_ in isolates from Clade IV were much higher than those from other three clades. Various *bla*_CTX-M_ subtypes (*bla*_CTX-M-65_, *bla*_CTX-M-55_, *bla*_CTX-M-27_, *bla*_CTX-M-14_, and *bla*_CTX-M-123_) with IS*Ecp1*, IS*903B*, IS*Vsa5*, and IS*1R* were found in ST17 isolates, especially Tn*1721* containing ΔIS*Ecp1*-*bla*_CTX-M-27_-IS*903B* in P1-like bacteriophage plasmids. These findings on the prevalent and genomic characterization for the *S*. Indiana multidrug-resistant ST17 clone in China, which have not been reported yet, provide valuable insights into the potential risk of this high-resistant clone.

**IMPORTANCE** Fluoroquinolones and cephalosporins are the primary choices for severe salmonellosis treatment. *S.* Indiana has become one of the most prevalent serovars in breeding poultry and poultry meats in China in recent years. ST17 was recognized as the leading epidemiological importance in *S.* Indiana because of its high-level resistance to the most of common antibiotics, including ciprofloxacin and ceftriaxone. However, the prevalence and phylogenomic characterization of ST17 isolates are unclear. Here, we did a retrospective screening on a large scale for *S.* Indiana in China, and performed its phylogenomic analysis. It was found that ST17 isolates had extensive spread in 15 provinces of China and became a multidrug-resistant clone. The international spread of the ST17 isolates was observed among several countries, especially China, the United Kingdom, and the United States. Our study emphasized the importance of surveillance of a high-resistant *S.* Indiana ST17 clone to combat its threat to public health.

## INTRODUCTION

Salmonellosis is one of the most common foodborne diseases worldwide, which poses a serious threat to public health (1, 2). Among more than 2,600 serovars of Salmonella, *S.* Indiana has extremely expanded to be one of the most prevalent serovars in China in recent years (3–6). The prevalence of *S.* Indiana in broiler chicken on livestock farms increased from 15% in 2010% to 70% in 2014 (7). Moreover, this pathogen has been found in patients with diarrhea in recent years (8, 9). Given the geographical area, *S.* Indiana has been reported in many provinces of China, including Shanghai, Shanxi, Henan, Hubei, Shandong, Hebei, Jiangsu, Anhui, Guangdong, Guangxi, Fujian, Jilin, and Inner Mongolia (6, 10–14), suggesting its wide geographical distribution.

Fluoroquinolones and cephalosporins are often used as the first-line drugs for salmonellosis treatments upon the failure of other therapeutic options. Ciprofloxacin and ceftriaxone are the representative antibiotics of fluoroquinolones and cephalosporins classes, respectively. However, ciprofloxacin and ceftriaxone co-resistance *S.* Indiana isolates have emerged, and become prevalent in retail meats and patients (8, 9). Furthermore, these isolates generally are multidrug-resistant (MDR) (6, 9, 14). We are now facing a formidable and growing menace from the emergence of MDR *S.* Indiana, and thus, it is vital to uncover epidemiological and evolutionary characterizations as well as antibiotic resistance mechanisms of this pathogen.

To date, some conventional methods such as PCR, pulse-field gel electrophoresis (PFGE), and multilocus sequence typing (MLST) have been used to explore the prevalence of *S*. Indiana (8, 12, 15). However, the conventional surveillance makes it difficult to obtain primary transmission dynamics of the expanded *S.* Indiana. Currently, whole-genome sequencing has been widely applied to reveal the evolutionary dynamics of bacteria (16–18). Therefore, a retrospective screening on a large scale was performed on 5, 287 serotyped Salmonella isolates from 16 provinces of China to elucidate the national prevalence of *S*. Indiana. The 171 genomes of *S*. Indiana isolates in this study together with those from other countries were used to give an insight into the phylogenomic structure and evolutionary characterizations of *S*. Indiana. Finally, the contribution of genetic factors to antibiotic resistance was explored in this pathogen.

## RESULTS AND DISCUSSION

### *S*. Indiana ST17 existed mainly in meats, patients, and healthy carriers in multiple provinces of China.

**FIG 1:**
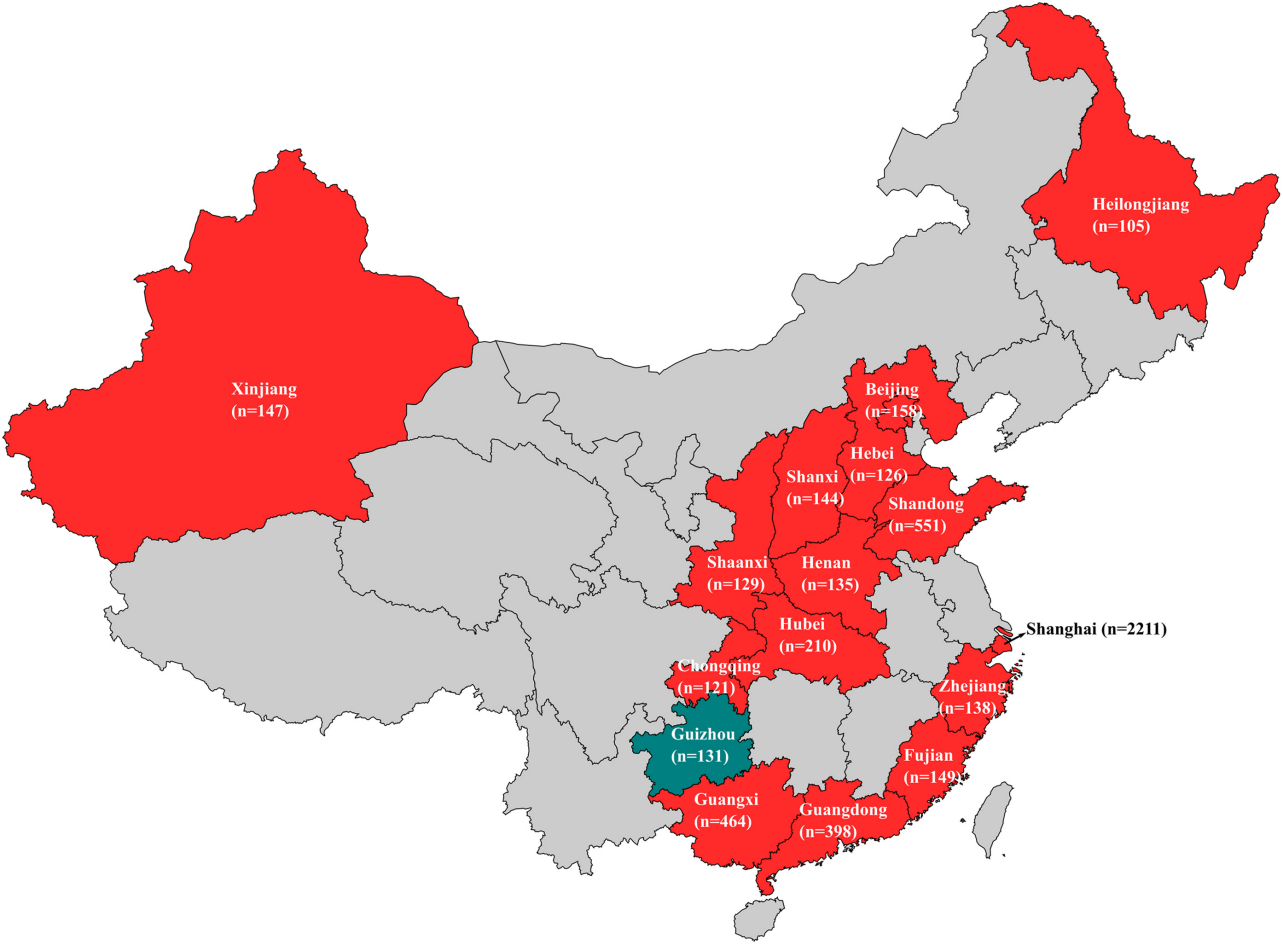
Domestic prevalence of *S*. Indiana ST17 isolates in China. The 16 provinces of a large scale to screen ST17 isolates are highlighted on the map, and the provinces where ST17 *S*. Indiana isolates were identified are indicated in red, and the province where ST17 isolate was not identified is indicated in blue green. The number of isolates obtained from each province is shown.

A total of 466 (8.81%) *S.* Indiana isolates were obtained from 5, 287 serotyped Salmonella isolates from foods, patients, healthy carriers, and environments in 15 of 16 provinces (Shanghai, Shandong, Guangdong, Guangxi, Hubei, Chongqing, Shaanxi, Shanxi, Heilongjiang, Beijing, Henan, Hebei, Zhejiang, Fujian, and Xinjiang) (Fig. 1). Among 466 isolates, 407 (87.3%) were identified to be ST17, and the rest (12.7%) were ST2040. The detection positive rate (87.3%) of ST17 isolates was lower than that in a previous study (100.0%) (9), which might result from the emergence of ST2040 isolates. To our knowledge, *S.* Indiana ST2040 has not been reported before, and further research will be needed on its prevalence and biological characteristics.

In this study, food samples (64.4%; 262/407) were the predominant sources of ST17 isolates (Table S1). It was further demonstrated that food samples were mainly composed of chicken, pork, duck, and marine products. A total of 186 (45.7%) isolates were recovered from chicken, accounting for the largest portion, and then duck (9.8%) and pork (3.7%) (Table S1), which suggested that chickens were the main vehicle of *S*. Indiana ST17 isolates. In addition to foods, 13.5% of ST17 isolates were found in healthy carriers (Table S1), which could be explained by its lower pathogenicity compared to *S*. Enteritidis and *S*. Typhimurium (19). However, healthy carriers with *S*. Indiana could not receive prompt treatment because they are asymptomatic. This was likely to accelerate the spread of *S*. Indiana isolates among humans. Besides, 15.2% of ST17 isolates were recovered from patients with diarrhea, which were mostly children or the elderly with low immunity.

### High-level multidrug resistances, including fluoroquinolones and cephalosporins resistance, occurred in *S*. Indiana ST17 from China.

**FIG 2:**
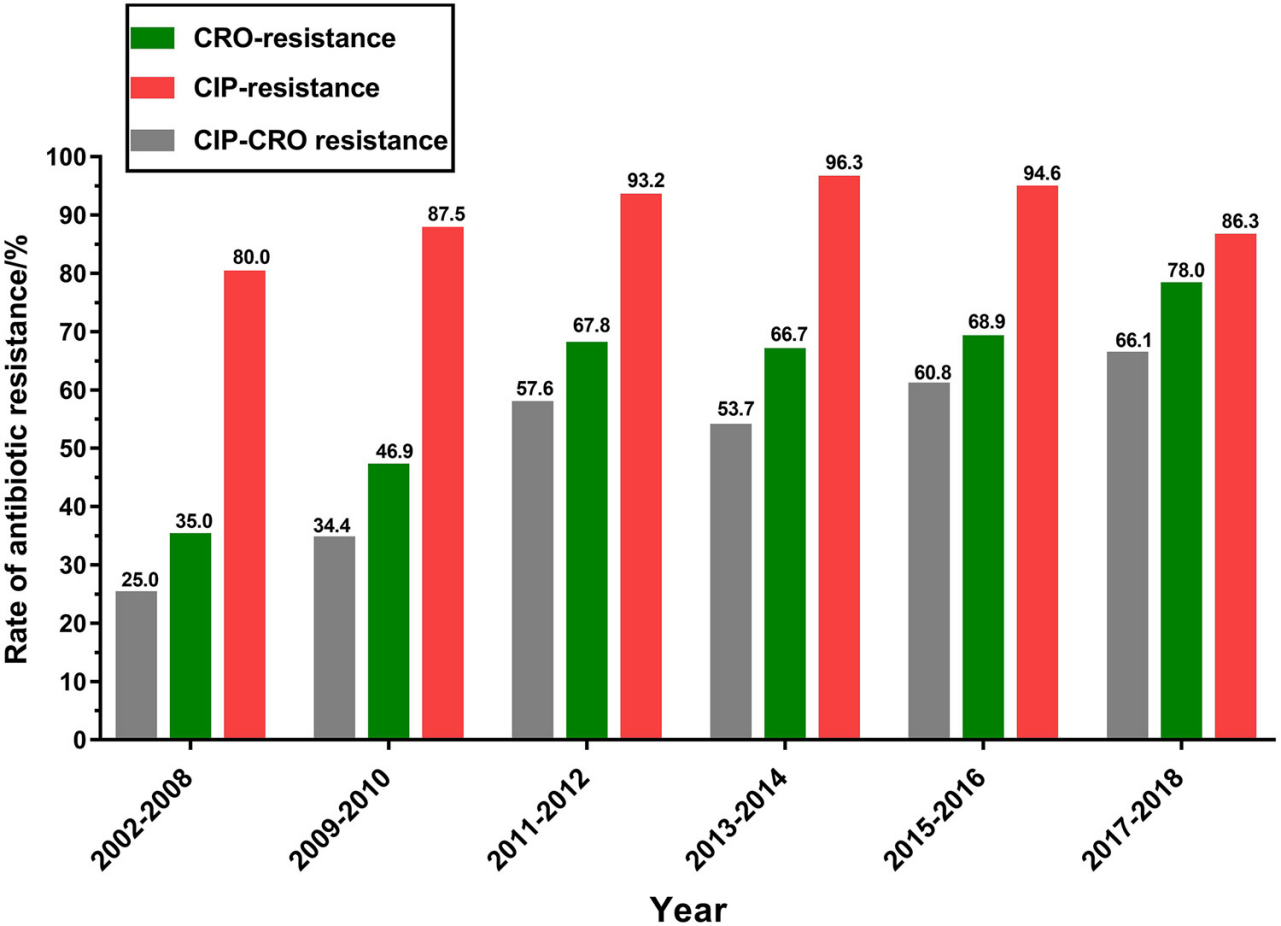
Prevalence of ciprofloxacin and ceftriaxone resistance in ST17 *S*. Indiana isolates between 2002 and 2018.

In this study, 366 (89.9%) of 407 *S.* Indiana ST17 isolates were resistant to ciprofloxacin, and 280 (68.8%) were resistant to ceftriaxone (Table S1). Furthermore, 235 (57.7%) isolates were concurrently resistant to both ciprofloxacin and ceftriaxone. There was an increase in ciprofloxacin resistance from 80.0% in 2002 to 2008 to 96.3% in 2013 to 2014 and dropped to 86.3% in 2017 to 2018 (Fig. 2). Ceftriaxone resistance was increased from 35.0% in 2002 to 2008 to 78.0% in 2017 to 2018. It was also observed that ciprofloxacin and ceftriaxone co-resistance was increased from 25.0% in 2002 to 2008 to 66.1% in 2017 to 2018 (Fig. 2). More importantly, a total of 372 (91.4%) ST17 isolates were identified to be MDR ones (Table S1). This high MDR rate of ST17 isolates was consistent with that (91.1%) in a previous study (9). Furthermore, 305 (74.9%) and 159 (39.1%) isolates were resistant, respectively, to at least five and seven classes of antibiotics. It was noted that an extensively drug-resistant *S*. Indiana ST17 isolate with resistance to 10 classes of antibiotics, including ciprofloxacin and ceftriaxone was obtained from a chicken carcass in 2012 in Guangdong, China in our previous study (20). Ciprofloxacin and ceftriaxone are the common drugs of choice for treating Salmonella infections (21). The high-level MDR in *S*. Indiana ST17 isolates, especially ciprofloxacin and ceftriaxone co-resistance would greatly increase the challenge of clinical treatments for patients.

Currently, *S*. Indiana isolates in other countries were relatively susceptible to most of the common antibiotics (22, 23). For example, *S*. Indiana isolates from Ethiopia exhibited susceptibility to ampicillin, amoxicillin-clavulanic acid, chloramphenicol, cephalothin, cefoxitin, streptomycin, and sulfamethoxazole-trimethoprim (22). Compared to those from other countries, it appeared that the high-level antibiotic resistance in *S*. Indiana was a unique phenomenon in China, which was likely to be caused by the widespread use of antibiotics in China. In 2013, the total amount of antibiotics used in China was about 162, 000 tons, approximately 160 times that of the United Kingdom, and 48% of which was used for human consumption, and the rest was shared by animals (24). Furthermore, the production yield of fluoroquinolones (including ciprofloxacin) and *β*-lactams (including ceftriaxone) in China was estimated to be 27, 300 and 34, 100 tons, respectively (24). Therefore, governmental regulations limiting the use of antimicrobial agents have been issued in China to reduce the potential threat of MDR bacteria to public health.

### International spread occurred in *S*. Indiana ST17 isolates.

To further determine the phylogenetic characteristics of *S*. Indiana isolates, phylogenomic analysis was performed on 171 genomes from China in this study and 470 genomes in the public databases from other 12 countries. A total of 3, 668 core SNPs were extracted from these 641 *S.* Indiana genome sequences, 2, 807 core SNPs of which were used to construct a maximum likelihood tree after 861 core SNPs in the recombinant regions were removed.

**FIG 3:**
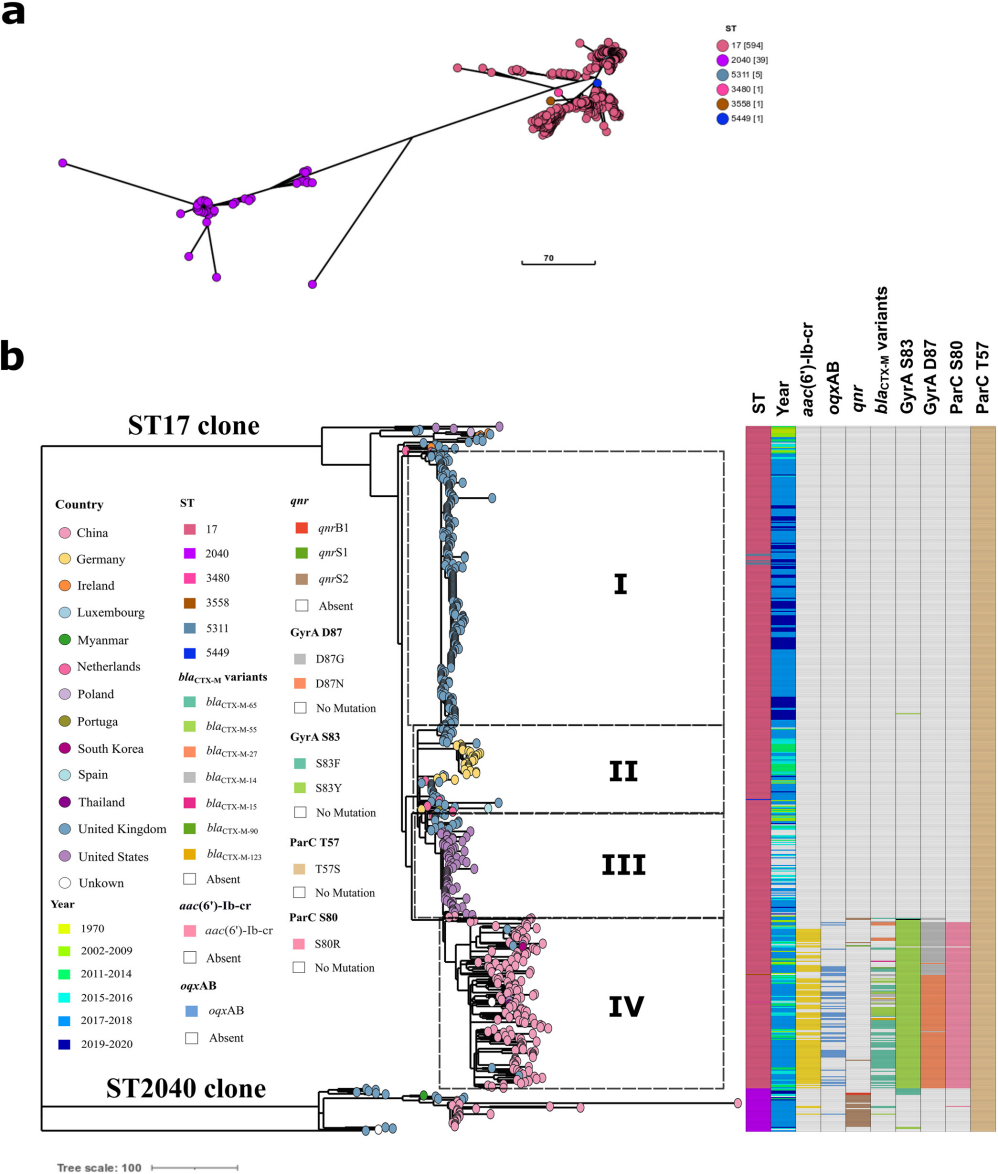
Evolutionary clones of *S*. Indiana international. (a) An unrooted phylogenetic tree f *S*. Indiana. (b) The maximum likelihood phylogenetic tree of *S*. Indiana isolates belonged to ST17 and ST2040 clones. Leaf nodes are colored by country (as shown in the inset legend). The colors of the isolates’ tips represent metadata columns, including MLST types, years of isolation, *bla*_CTX-M_ variants, PMQR genes (*oqx*AB, *qnr,* and *aac*[6’]-Ib-cr), and mutations in QRDR (GyrA S83/D87 and ParC T57/S80) (as shown in the inset legend). *bla*_CTX-M_ variants columns included *bla*_CTX-M-65_, *bla*_CTX-M-55_, *bla*_CTX-M-14_, *bla*_CTX-M-27_, *bla*_CTX-M-123_, *bla*_CTX-M-15_ and *bla*_CTX-M-90_ (as shown in the inset legend). Light gray shading shows Clade I, II, III, and IV.

A total of six sequence types (STs) (ST17, ST2040, ST3480, ST3558, ST5311, and ST5449) were identified in these 641 *S.* Indiana isolates, of which ST17 (*n* = 594) and ST2040 (*n* = 39) were the major types (Fig. 3a). Compared to ST2040 isolates, ST17 isolates were widely disseminated in 13 countries tested across Europe, North America, and Southeast Asia, and persisted for many years (1970 to 2020) (Fig. 3b and Fig. S1). Furthermore, it was found in Fig. 3b that ST17 and ST2040 clones had emerged, which could be proved by their long genetic distance.

Phylogenomic results indicated ST17 isolates might early emerge in the United States because they were the base of the phylogenetic tree, and then were evolved in Ireland and the United Kingdom, and finally separated into four clades (I, II, III, and IV) internationally (Fig. 3b). Clade I was composed of 250 isolates from the United Kingdom between 2014 and 2020. Clade II was a mixed cluster, which was composed of isolates from the Netherlands, Germany, and the United Kingdom in European, suggesting that their genetic relationships were close. Clade III was composed of isolates from the United States and the United Kingdom, and their evolutionary path implied that a major introduction might occur in the United States from the United Kingdom, and then nationwide spread. Furthermore, ST17 isolates from China during 2006 to 2018 fell into Clade IV, and four human isolates from the United Kingdom also belonged to Clade IV (Fig. 3b and Fig. S2). It was quite possible that the persons traveled to China, returned to the United Kingdom, and then became sick. Other sources of isolates from different countries were found in Clade IV including chicken (South Korea, *n* = 1), porcini Mushrooms (United States, *n* = 1), and dried chill (Thailand, *n* = 1) (Fig. S2), suggesting their close genetic relationship to those from China.

### Mutations in GyrA and ParC as well as PMQR genes frequently happened in *S*. Indiana ST17 from China.

It was observed in isolates from China that mutations in GyrA-ParC (84.8%), PMQR genes of *aac*(6′)-Ib-cr (62.0%) and *oqx*AB (29.8%) were frequently identified. Mutations in QRDR of GyrA were commonly found as S83F/D87N (57.3%) and S83F/D87G (24.6%), followed by S83F (1.8%) and D87G (1.2%). Mutations in ParC were commonly found as T57S/S80R (*n* = 83.0%), followed by T57S (17.0%). Compared with S. Enteritidis and *S.* Typhimurium (25, 26), *S*. Indiana isolates possessed more site mutations in GyrA and ParC. Furthermore, it was found that rates of mutations in GyrA and ParC in *S*. Indiana isolates from China were significantly higher (*P* < 0.05) than those from the United Kingdom, United States, and Germany (Table S3). The mutations in GyrA-ParC were identified in 145 (84.8%) isolates from China, but these mutations were only found in 12 (3.7%) and 2 (2.4%) isolates from the United Kingdom and the United States, respectively (Table S3). In addition, the detection positive rate of *aac*(6′)-Ib-cr and *oqx*AB in *S*. Indiana isolates from China were significantly higher (*P* < 0.05) than those from the United Kingdom, United States, and Germany. Mutations in QRDR and PMQR genes were mainly responsible for the decreased susceptibility to ciprofloxacin in Gram-negative bacteria (27). These results showed that mutations in GyrA and ParC as well as PMQR genes were frequently identified in *S*. Indiana ST17 from China, which could be a reason for the high-level ciprofloxacin resistance in this study.

It was found in Fig. 3b that a mutation also occurred in ParC (T57S) in ST2040 isolates. It was interesting that the mutation in ParC (T57S) was identified in all 641 *S.* Indiana isolates tested including ST17 and ST2040 isolates in this study from 13 countries across the years of 1970 to 2020, suggesting that there might be a natural mutation site in *S.* Indiana.

### High-level MICs of ciprofloxacin against ST17 isolates resulted from the combination of mutations in GyrA-ParC and PMQR genes.

**FIG 4:**
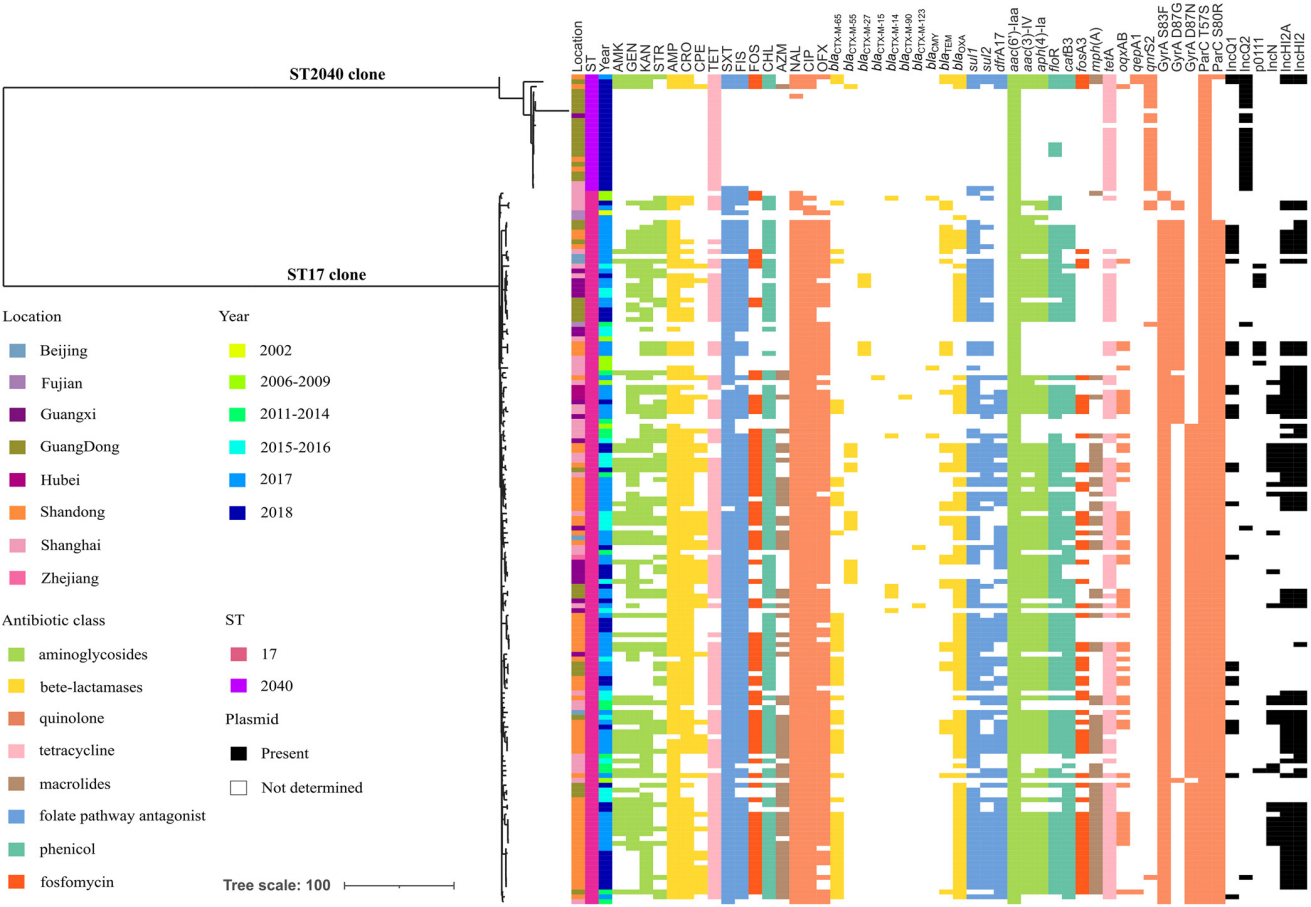
The phylogenetic tree of ST17 *S*. Indiana isolates in China. Leaf nodes are colored by provinces (see the key). The colors of isolate tips represent years of isolation and metadata columns, including *bla*_CTX-M_ variants, PMQR genes (*oqx*AB, *qnr,* and *aac*[6’]-Ib-cr) and mutations in QRDR (GyrA S83/D87 and ParC T57/S80), and blocks colored by antibiotic class.

As shown in Fig. 4, there were multiple site mutations in QRDR accompanied by a dramatic clonal expansion in ST17 isolates in China through analysis of a reconstructed phylogenetic tree. In particular, a single mutation occurred in GyrA (S83F or D87N) in the base isolates of the ST17 clone, followed by double mutations in GyrA (S83F/D87N or S83F/D87G) with an additional mutation in ParC (S80R). Similarly, phylogenomic analysis indicated that the early mutation occurred in GyrA (S83F), followed by a mutation in ParC (S80I) with a dramatic expansion in Salmonella Kentucky ST198 (28). These findings implied that the increase in site mutations in GyrA and ParC might contribute to the expanded dissemination of *S*. Indiana ST17 through enhancing its antibiotic resistance.

The mutations in GyrA and ParC were constructed in a susceptible wild-type isolate (*S*. Indiana SJTUF14139) through homologous recombination yielding mutants of PML1 (GyrAS83F), PML2 (GyrAD87N), PML3 (GyrAS83F/D87N), and PML4 (GyrAS83F/D87N-ParCS80R) to examine their role in ciprofloxacin resistance formation (Table S6). The results from growth curves showed that mutations in GyrA and ParC had no significant effect on the growth rate of PML1-4 (Fig. S4). Furthermore, a single mutation in GyrA (S83F or D87N) or double mutations in GyrA (S83F/D87N) resulted in an increase in MIC of ciprofloxacin from 0.015 μg/mL to 0.25 μg/mL. Triple mutations (GyrAS83F/D87N-ParCS80R) in PML4 resulted in a higher MIC of ciprofloxacin (8 μg/mL), which exceeded the breakpoint resistance value (1 μg/mL) according to the CLSI 2019 profile.

Our previous study indicated that the MICs of ciprofloxacin against *S*. Indiana were generally higher than 64 μg/mL (15), and it seemed that mutations in GyrA and ParC were not the only reason for the high-level MIC of ciprofloxacin. Therefore, a recombinant plasmid pMD*oqx*AB was constructed by cloning PMQR gene *oqx*AB into pMD19-T vector, which was then introduced into PML1-4 to yield the mutants PML5 (GyrAS83F-pMD*oqx*AB), PML6 (GyrAD87N-pMD*oqx*AB), PML7 (GyrAS83F/D87N-pMD*oqx*AB) and PML8 (GyrAS83F/D87N-ParCS80R-pMD*oqx*AB). Compared with the wild-type strain SJTUF14139, the growth rates of PML5, PML6, PML7, and PML8 were lower (Fig. S4), which might result from fitness cost through the introduction of pMD*oqx*AB. Compared with those in PML1-4, the MICs of ciprofloxacin against PML5-8 were increased by 4 to 8 times. The MICs of ciprofloxacin against PML5, PML6, and PML7 reached at least 1 μg/mL, which indicated that mutations in GyrA (S83F and/or D87N) combined with *oqx*AB could make the MIC of ciprofloxacin reach the breaking resistance point (1 μg/mL). Moreover, the MIC of ciprofloxacin against PML8 (GyrAS83F/D87N-ParCS80R-pMD*oqx*AB) reached 32 μg/mL, which was 32 times higher than the breaking resistance point (1 μg/mL). These findings indicated that high-level MICs of ciprofloxacin against ST17 isolates resulted from the combined action of mutations in GyrA-ParC and PMQR genes.

### Multiple antibiotic resistance genes (ARGs) appeared on IncHI2-IncHI2A plasmids in ST17 isolates.

**FIG 5:**
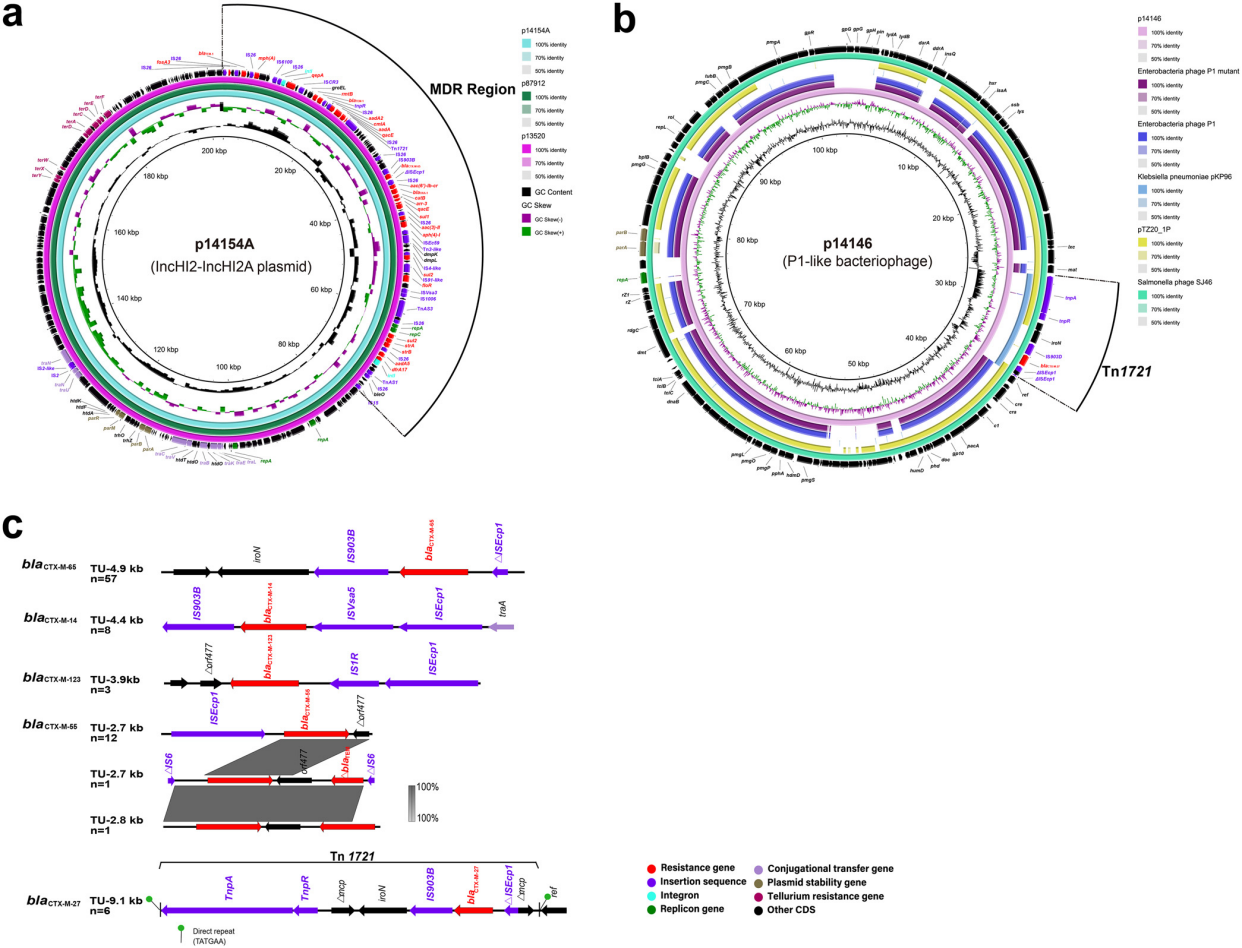
Plasmid sequence characterization and genetic arrangement in *S*. Indiana. (a) Sequence comparison of plasmid p14154A (Accession number CP064667.1) in this study, p13520 (Accession number CP041182) and p87912 (Accession number CP041180). (b) Sequence comparison of plasmid p14146 (Accession number CP064673.1) in this study, Enterobacteria phage P1 (Accession number NC_005856.1), Enterobacteria phage P1 (Accession number AF234172.1), Klebsiella pneumonia pKP96 (Accession number EU195449.1), Salmonella phage SJ46 (Accession number KU760857.1), and Escherichia coli pTZ20_1P (Accession number MN510447.1). (c) Genetic arrangements of *bla*_CTX-M-65_, *bla*_CTX-M-55_, *bla*_CTX-M-14_, *bla*_CTX-M-123_ and *bla*_CTX-M-27_. Areas shaded in gray indicate homologies between the corresponding genetic loci.

It was found that there were diverse types of plasmids in ST17 isolates, which were IncHI2-IncHI2A, IncN, IncQ1, IncQ2 and P1 phage-like types. In this study, the IncHI2-IncHI2A plasmid was the most frequently observed in *S*. Indiana isolates in China, accounting for 68.4%. Therefore, an IncHI2-IncHI2A plasmid (p14154A) was extracted from the SJTUF14154 isolate and further sequenced for analysis. It was found that p14154A was highly similar to our previous reported plasmid p87912 (Accession number CP041180; 100% coverage; 100% identity) (20) and p13520 (Accession number CP041182; 100% coverage; 99.2% identity) (15) (Fig. 5a). The hosts of these three plasmids were from different places (p14154A, Shandong; p13520, Shanghai; p87912, Guangdong), suggesting that these plasmids were likely to evolve from the same ancestor.

A total of 26 ARGs were found to be located in the multidrug resistance region (MRR) with a size of ~75 kbp in plasmid p14154A. In our previous study, an IncHI2-IncHI2A plasmid p87912 was transferred from the donor to the recipient E. coli DH5α accompanied by the transfer of MDR phenotype (20), which indicated that IncHI2-IncHI2A plasmids bearing multiple ARGs played an important role in MDR in *S.* Indiana.

It was found that ARGs of *bla*_CTX-M-65_, *mph*A, *qep*A, *oqx*AB, *bla*_OXA-1_, *fos*A3, and *rmt*B interspersed with different ISs and transposons (Tns) (IS*26*, IS*Ecp1*, IS*CR3*, IS*Vsa3*, IS*1006*, IS*903B*, Tn*1721*, and Tn*3-*like) in plasmid p14154A. The genomic rearrangements and genetic exchanges were always mediated by mobile elements (29). For example, IS*26* is the common mobile element, which is the major component of antibiotic resistance determinants. Replicative transposition or homologous recombination on conservative regions frequently occurred in IS*26*, and then created a new MRR (29, 30). The current study and previous studies (15, 20, 30) showed that most of MRRs had a mosaic structure bound at both ends by fragments of mobile elements, which implied that the formation of an MRR in *S*. Indiana ST17 was involved in the acquisition and stepwise integration of multiple ARGs determinants through homologous recombination or transposition of mobile elements.

### P1-like bacteriophage plasmids bearing Tn*1721*-*bla*_CTX-M-27_ emerged in ST17 isolates.

In this study, nine ST17 isolates were identified to possess P1-like replicon genes, and six were found to be positive for ESBL gene *bla*_CTX-M-27_, which was seldomly found in other isolates. Therefore, a plasmid p14146 was extracted from the SJTUF14146 isolate and sequenced for analysis, which was proved to be a P1-like bacteriophage plasmid, sharing the high similar sequence structure with Enterobacteria phage P1 (Fig. 5b). Furthermore, a Tn*1721* unit harboring *bla*_CTX-M-27_ was integrated into p14146, and it was flanked by two 6 bp direct repeat sequences (TATGAA), suggesting a transposition event occurred (Fig. 5c). Gene *bla*_CTX-M-27_ in the Tn*1721* unit was linked to the transposable element ΔIS*Ecp1* upstream and IS*903B* downstream. Our study and previous studies (31, 32) indicated that *bla*_CTX-M-27_ was frequently located in Tn*1721* transposon unit in *S*. Indiana ST17, which implied that Tn*1721* might accelerate the horizontal transfer of *bla*_CTX-M-27_ gene through transposition.

### Multiple *bla*_CTX-M_ variants existed in ST17 isolates from China.

It was demonstrated in Table S3 that ESBL genes *bla*_OXA-1_, *bla*_CTX-M_ and *bla*_TEM-1B_ were identified, respectively, in 106, 89 and 36 isolates from China (Table S3). A total of seven *bla*_CTX-M_ variants were identified in this study, which were *bla*_CTX-M-65_, *bla*_CTX-M-55_, *bla*_CTX-M-14_, *bla*_CTX-M-27_, *bla*_CTX-M-123_, *bla*_CTX-M-15_, and *bla*_CTX-M-90_. Except that two *bla*_CTX-M_ variants, including *bla*_CTX-M-65_ (*n* = 2; ST17) and *bla*_CTX-M-14_ (*n* = 3; ST17 and ST3358) were identified in the isolates from other countries, the remaining variants were identified in ST17 (*n* = 86) and ST2040 isolates (*n* = 3) from China. Among these variants, *bla*_CTX-M-65_ (*n* = 55) was the most common one in ST17 isolates, followed by *bla*_CTX-M-55_ (*n* = 14), *bla*_CTX-M-14_ (*n* = 6), *bla*_CTX-M-27_ (*n* = 6), *bla*_CTX-M-123_ (*n* = 3), *bla*_CTX-M-15_ (*n* = 1) and *bla*_CTX-M-90_ (*n* = 1). The predominant subgenotype of *bla*_CTX-M_ varied in different Salmonella serotypes. These included *bla*_CTX-M-65_ in *S*. Indiana in this study, *bla*_CTX-M-55_ in S. Enteritidis (33), *bla*_CTX-M-9_ in *S*. Kentucky (34), and *bla*_CTX-M-14_ in *S.* Typhimurium (35). In particular, these *bla*_CTX-M_ genes were generally located on the plasmids, which might contribute to the rapid expansion of ceftriaxone resistance through conjugation among Enterobacteriaceae (36–38).

As shown in Fig. 5c, gene *bla*_CTX-M-65_ was frequently linked to the transposable elements ΔIS*Ecp1* upstream and IS*903B* downstream, and this transposon unit was about 4.9 kbp. It was found in p14154A that the truncated ΔIS*Ecp1* upstream of *bla*_CTX-M-65_ was interrupted by IS*26*. On the contrary, a complete IS*Ecp1* element was found in the transposon units of *bla*_CTX-M-14_ and *bla*_CTX-M-123_, inserting the upstream of a complete element IS*Vsa5* and IS*1R*. The difference in the transposon unit between *bla*_CTX-M-14_ and *bla*_CTX-M-123_ was that downstream of the former was IS*903B*, but that of the latter was Δ*orf*477. Gene *bla*_CTX-M-55_ was frequently found to be linked to IS*Ecp1* upstream and Δ*orf*477 downstream. The horizontal transfer of ARGs is found to be mediated by mobile genetic elements like transposons, insertion sequences, and integrons (39). Insertion sequences are able to move themselves and ARGs to new locations in the same or different DNA molecules such as chromosomes and plasmids (29). A previous study demonstrated that IS*Ecp1* could move *bla*_CTX-M-2_ from the chromosome to the plasmid in Kluyvera ascorbata through transposition (40). The current study showed that IS*Ecp1* was the most common mobile element associated with *bla*_CTX-M_ genes, which might play a key role in the horizontal transfer of *bla*_CTX-M_ genes in *S*. Indiana.

### Conclusion.

Characterization on the large-scale emergence and international spread of an *S*. Indiana ST17 clone was systematically carried out in this study, which has not been reported before. High-level MDR including ciprofloxacin and ceftriaxone resistance was found in the ST17 clone from China, which might be explained by their high mutations in GyrA and ParC as well as multiple ARGs, including PMQR genes and ESBL genes from plasmids. Our study suggests that necessary strategies are warranted to prevent the further dissemination of this high-risk MDR ST17 clone. Therefore, China government has issued regulations to prohibit excessive use of antibiotics in livestock farms and hospitals, and to strengthen education and publicity on bacterial resistance.

## MATERIALS AND METHODS

### Bacterial isolates.

A total of 5, 287 Salmonella isolates from foods, patients, and environments in 16 provinces of China were collected between 2002 and 2018. Food sources included chicken, pork, duck, marine products, and frozen food. Human sources included the stool and blood of health checkers, outpatients, and inpatients in hospitals for diarrhea treatment. Environment sources included municipal sewage and poultry feces. All above Salmonella isolates were identified via API20E test strips (bioMérieux, France) and serotyped via commercial antiserum (Statens Serum Institute, Copenhagen, Denmark) according to the manufacturer’s guidelines.

### Antimicrobial susceptibility testing.

Antimicrobial susceptibility testing was performed on *S*. Indiana isolates using the agar dilution method provided by the Clinical and Laboratory Standard Institute (41) and the European Committee on Antimicrobial Susceptibility Testing (42). The following antibiotics were selected: amikacin (AMK), ampicillin (AMP), ceftiofur (TIO), ceftriaxone (CRO), cefoxitin (FOX), cefepime (CPE), nalidixic acid (NAL), ciprofloxacin (CIP), ofloxacin (OFX), chloramphenicol (CHL), kanamycin (KAN), gentamicin (GEN), streptomycin (STR), tetracycline (TET), sulfamethoxazole/trimethoprim (SXT), sulfisoxazole (FIS), azithromycin (AZM), fosfomycin (FOS), meropenem (MEM), and imipenem (IMP). Bacterial susceptibility to colistin (CT) was performed with broth microdilution method recommended by the European Committee on Antimicrobial Susceptibility Testing. Escherichia coli ATCC 25922 and Enterococcus faecalis ATCC 29212 were used as quality control strains.

### Whole-genome sequencing.

Bacterial cells were grown in 250 mL liquid Luria-Bertani (LB) medium overnight at 37°C, with agitation at 200 rpm. Genomic DNA was extracted from *S.* Indiana isolates using the QIAamp DNA minikit (Qiagen, CA). The sequencing service was provided by the Personal Biotechnology Company (Shanghai, China). The whole-genome sequencing (WGS) was performed by using the Illumina MiSeq platform (Illumina, San Diego, CA, USA). The plasmid sequences were extracted from the whole-genome sequences using the PacBio RS II system (Pacific Biosciences, Menlo Park, CA, USA). For the Illumina MiSeq platform, a 400-bp DNA library was constructed and sequenced by the paired-end sequencing mode. Data generated from the Illumina MiSeq platform was assembled by SPAdes (43). For the PacBio RS II platform, a 10-kbp DNA library was constructed and sequenced using single-molecule real-time (SMRT) sequencing technology. Sequence data from the PacBio RS II platform was assembled using the Canu software (44). Finally, the consensus genome sequence was determined using the Pilon software (45). Sequence data of read numbers, total bases, GC contents, and Q20/30 rates was available in Table S7 in the supplemental material.

Annotation of the genome was performed using RAST (46), BLASTn, and BLASTp programs. The encoding genes in the genome were predicted by Glimmer (47) and GeneMarkS (48). The tRNAs, rRNAs, and repeated sequences in the genome were predicted by tRNAscan-SE v2.0 (http://trna.ucsc.edu/software/), Barrnap (https://github.com/tseemann/barrnap), and Tandem Repeats Finder (http://tandem.bu.edu/trf/trf.html), respectively.

### Antimicrobial resistance determinants.

ResFinder 4.1 was used to identify ARGs and chromosomal mutations mediating antibiotic resistance in the genome (49). MLST 2.0 was used to identify the STs of bacteria (50). Plasmidfinder was used to identify replicon types of plasmids (51). ISfinder (https://www-is.biotoul.fr/) was used to analyze the IS and transposons in the genome.

### Phylogenomic analysis.

A total of 171 genomes in this study, including a reference genome of *S*. Indiana SJTUF87912v2 (Accession number CP041179.1) with 470 genomes of *S*. Indiana isolates available from the Enterobase database (http://enterobase.warwick.ac.uk/species/index/senterica, August 2021) were used for phylogenomic analysis. Single-nucleotide polymorphisms (SNPs) were extracted using Snippy (https://github.com/tseemann/snippy) to generate core genomic alignment. Gubbins (52) was then used to remove recombination regions. The core SNP alignment was used to generate a maximum-likelihood phylogeny using RAxML v8.1.23 (53) with the GTR nucleotide substitution model. Furthermore, 100 random bootstrap replicates were conducted to assess the node support. The phylogenetic tree was visualized together with metadata using Microreact v5.99.0 (54).

### Gene cloning and generation of *gyr*A and *par*C mutants.

The *oqx*AB, *gyr*A and *par*C fragments were amplified by PCR using primers (Table S4), and then cloned into pMD19-T vector, yielding recombinant plasmids pMD19-T-*oqx*AB and pMD19-T-*gyr*A/*par*C. Recombinant plasmid pMD19-T-*oqx*AB was transformed into E. coli DH5α using electroporation. A suicidal vector pRE112 was used to construct *gyr*A and *par*C mutants through homologous recombination (55). Point mutations on plasmids pMD19-T-*gyr*A/*par*C were constructed using QuikChange Lightning Site-Directed Mutagenesis kit (Agilent, USA). The above-constructed plasmids were then ligated to pRE112, yielding recombinant pRE112-*gyr*A/*par*C plasmids. Then, the obtained pRE112-*gyr*A/*par*C recombinant plasmids were transformed into an *S*. Indiana wild-type strain SJTUF14139 with a pKOBEG plasmid in advance. Along with inducing by arabinose and saccharose, wild *gyr*A/*par*C sequence bases were replaced with point mutations through homologous recombination. The amino acid substitutions of GyrA and ParC were analyzed and aligned using BLASTP (https://blast.ncbi.nlm.nih.gov/Blast.cgi?PROGRAM=blastp&PAGE_TYPE=BlastSearch&BLAST_SPEC=&LINK_LOC=blasttab&LAST_PAGE=blastn) to confirm the specific mutations. The above plasmids used in this study for constructing mutants could be found in Table S5 in the supplemental material.

### Growth curves.

The wild-type strain SJTUF14139 and its mutants were inoculated into 30 mL fresh LB broth overnight at 37°C and 200 rpm. Then, bacterial suspensions were adjusted to an OD_600_ nm of approximately 0.1. The growth curves of bacteria were measured at regular time intervals (1 h) by a Bioscreen C Analyzer (TYPE FP-1100-C, Oy Growth Curves Ab Ltd.). Experiments were performed in three independent assays.

### Data availability.

The genome sequence data were available from BioProject ID: PRJNA641453, and a reference genome SJTUF87912v2 was deposited in the NCBI database under the accession number CP041179.1. The plasmid sequences of p14154A and p14146 had been deposited in the NCBI database under the accession numbers CP064667.1 and CP064673.1, respectively. Phylogenetic analysis of *S.* Indiana isolates worldwide and in China with metadata were available at Microreact URLs https://microreact.org/project/tpf7vkSgqDj18ALxtiuHyw and https://microreact.org/project/6N4ie9oT7SVqsK3qWm9jLa.
