# Supplementary material for: Phylogenomic Analysis of Salmonella enterica Serovar Indiana ST17, an Emerging Multidrug-Resistant Clone in China
Source: Microbiol Spectr. 2022 Jul 5;10(4):e00115-22. doi: 10.1128/spectrum.00115-22 (PMC9430114; doi:10.1128/spectrum.00115-22)
Supplement: Supplemental file 1 — Supplemental material. Download spectrum.00115-22-s0001.pdf, PDF file, 1.0 MB [file spectrum.00115-22-s0001.pdf]

Supplementary Data

Table S1 Antimicrobial resistance in 407 *S*. Indiana ST17 isolates from humans, foods and environment settings in China

Antimicrobials	Resistant isolates (No. / ratio %)										Total (n=407)
	Humans			Foods						Environment (n=28)	
	Infected(n=62)	Carrier(n=55)	Total(n=117)	Chicken(n=186)	Duck(n=40)	Pork(n=15)	Marine products(n=12)	Other(n=9)	Total(n=262)		
***β*-Lactams**											
Ampicillin	41/66.1	43/78.2	84/71.8	155/83.3	34/85.0	9/60.0	9/75.0	7/77.8	214/81.7	24/85.7	322/79.1
Ceftriaxone	56/90.3	32/58.2	88/75.2	130/69.9	27/67.5	5/33.3	5/41.7	4/44.4	171/65.3	21/75.0	280/68.8
Cefepime	46/74.2	17/30.9	63/53.8	45/24.2	13/32.5	2/13.3	2/16.7	2/22.2	64/24.4	5/17.8	132/32.4
**Aminoglycosides**											
Amikacin	9/14.5	2/3.6	11/9.4	65/34.9	5/12.5	3/20.0	5/41.7	2/22.2	80/30.5	9/32.1	100/24.6
Gentamicin	37/59.7	24/43.6	61/52.4	103/55.4	17/42.5	6/40.0	7/58.3	3/33.3	136/51.9	15/53.6	212/52.1
Streptomycin	38/61.3	16/29.1	54/46.2	70/37.6	10/25.0	6/40.0	4/33.3	2/22.2	92/35.1	17/60.7	163/40.0
Kanamycin	33/53.2	19/34.5	52/44.4	111/59.7	20/50.0	12/80.0	6/50.0	2/22.2	151/57.6	20/71.4	223/54.8
**Quinolones**											
Nalidixic acid	61/98.4	48/87.3	109/93.2	171/91.9	34/85.0	11/73.3	11/91.7	9/100.0	236/90.1	28/100.0	373/91.6
Ciprofloxacin	60/96.8	42/76.4	102/87.2	172/92.5	33/82.5	11/73.3	11/91.7	9/100.0	236/90.1	28/100.0	366/89.9
Ofloxacin	61/98.4	41/74.5	102/87.2	145/78.0	20/50.0	10/66.7	10/83.3	8/88.9	193/73.7	24/100.0	319/78.4
**Tetracyclines**											
Tetracycline	41/66.1	43/78.2	84/71.8	144/77.4	31/77.5	10/66.7	5/41.7	5/55.6	195/74.4	19/67.8	298/73.2
**Sulphamethoxazole**											
Sulfisoxazole	57/91.9	52/94.5	109/93.2	139/74.7	31/77.5	7/46.7	7/58.3	5/55.6	189/72.1	26/92.8	324/79.6
**Folate pathway antagonists**											
Trimethoprim-sulfamethoxazole	62/100.0	55/100.0	117/100.0	163/87.6	32/80.0	11/73.3	11/91.7	9/100.0	226/86.3	28/100.0	371/91.2
**Phenicols**											
Chloramphenicol	44/71.0	30/54.5	74/63.2	131/70.4	29/72.5	5/33.3	5/41.7	5/55.6	175/66.8	21/75.0	270/66.3
**Macrolides**											
Azithromycin	9/14.5	3/5.5	12/10.3	80/43.0	3/7.5	1/6.7	1/8.3	0/0.0	85/32.4	13/46.4	110/27.0
**Fosfomycins**											

Fosfomycin	33/53.2	8/14.5	41/35.0	101/54.3	12/30.0	5/33.3	5/41.7	5/55.6	128/48.9	11/39.3	180/44.2
**Lipopeptides**											
Colistin	0/0.0	0/0.0	0/0.0	0/0.0	0/0.0	0/0.0	0/0.0	0/0.0	0/0.0	0/0.0	0/0.0
**Carbapenems**											
Meropenem	0/0.0	0/0.0	0/0.0	0/0.0	0/0.0	0/0.0	0/0.0	0/0.0	0/0.0	0/0.0	0/0.0
Imipenem	0/0.0	0/0.0	0/0.0	0/0.0	0/0.0	0/0.0	0/0.0	0/0.0	0/0.0	0/0.0	0/0.0
≥3(MDR)	61/98.4	49/89.1	110/94.0	169/90.9	35/87.5	14/93.3	10/83.3	7/77.8	235/89.7	27/96.4	372/91.4
≥5	50/80.6	34/61.8	84/71.8	143/76.9	28/70.0	12/80.0	7/58.3	6/66.7	196/74.8	25/89.3	305/74.9
≥7	22/35.5	4/7.3	26/22.2	101/54.3	11/27.5	6/40.0	2/16.7	2/22.2	122/46.6	11/39.3	159/39.1

Table S2 Antimicrobial resistance in 407 *S*. Indiana ST17 isolates from different provinces in China

Antimicrobials	resistant isolates (No. / ratio %)										
	North				South						Total (n=407)
	Shandong (n=76)	Beijing (n=7)	Other (n=9)	Total (n=92)	Shanghai (n=164)	Guangdong (n=48)	Guangxi (n=70)	Hubei (n=16)	Other (n=17)	Total (n=315)	
***β*-Lactams**											
Ampicillin	76/100.0	7/100.0	9/100.0	92/100.0	135/77.1	57/67.1	47/67.1	15/93.8	11/64.7	230/73.0	322/79.1
Ceftriaxone	75/98.7	6/85.7	7/77.8	88/95.7	96/54.9	39/45.9	38/44.3	12/75.0	8/47.1	192/61.0	280/68.8
Cefepime	30/39.5	2/28.6	5/55.6	37/40.2	27/15.4	13/15.3	22/31.4	4/25.0	3/17.6	95/30.2	132/32.4
**Aminoglycosides**											
Amikacin	45/59.2	3/42.9	4/44.4	52/56.5	34/19.4	12/14.1e	5/7.1e	6/37.5	0/0.0	48/15.2e	100/24.6
Gentamicin	60/78.9	4/57.1	7/77.8	71/77.2	66/37.7	32/37.6	29/41.4	15/93.8	9/52.9	141/44.8	212/52.1
Streptomycin	39/51.3	3/42.9	4/44.4	46/50.0	66/37.7	23/27.1	21/30.0	8/50.0	9/52.9	117/37.1	163/40.0
Kanamycin	66/86.8	5/71.4	7/77.8	78/84.8	96/54.9	32/37.6	26/37.1	12/75.0	5/29.4	145/46.0	223/54.8
**Quinolones**											
Nalidixic acid	76/100.0	7/100.0	9/100.0	92/100.0	173/98.9	57/67.1	64/91.4	16/100.0	14/82.4	281/89.2	373/91.6
Ciprofloxacin	76/100.0	7/100.0	9/100.0	92/100.0	168/96.0	58/68.2	57/81.4	16/100.0	15/88.2	274/87.0	366/89.9
Ofloxacin	74/97.4	6/85.7	9/100.0	89/96.7	158/90.3	44/51.8	52/74.3	8/50.0	15/88.2	230/73.0	319/78.4
**Tetracyclines**											
Tetracycline	59/77.6	5/71.4	9/100.0	73/79.3	96/54.9	76/89.4	57/81.4	7/43.8	9/52.9	225/71.4	298/73.2
**Sulphamethoxazole**											
Sulfisoxazole	76/100.0	6/85.7	9/100.0	91/98.9	121/69.1	51/60.0	61/87.1	15/93.8	15/88.2	233/74.0	324/79.6
**Folate pathway antagonists**											
Trimethoprim-sulfamethoxazole	75/98.7	6/85.7	9/100.0	90/97.8	168/96.0	55/64.7	69/98.6	14/87.5	15/88.2	281/89.2	371/91.2
**Phenicols**											
Chloramphenicol	74/97.4	6/85.7	7/77.8	87/94.6	98/56.0	48/56.5	39/55.7	15/93.8	11/64.7	183/58.1	270/66.3
**Macrolides**											

Azithromycin	59/77.6	2/28.6	1/11.1	62/67.4	46/26.3	12/14.1	5/7.1	7/43.8	1/5.9	48/15.2	110/27.0
**Fosfomycins**											
Fosfomycin	71/93.4	6/85.7	6/66.7	83/90.2	78/44.6	22/25.9e	12/17.1e	5/31.3e	7/41.2	97/30.8	180/44.2
**Lipopeptides**											
Colistin	0/0.0	0/0.0	0/0.0	0/0.0	0/0.0	0/0.0	0/0.0	0/0.0	0/0.0	0/0.0	0/0.0
**Carbapenems**											
Meropenem	0/0.0	0/0.0	0/0.0	0/0.0	0/0.0	0/0.0	0/0.0	0/0.0	0/0.0	0/0.0	0/0.0
Imipenem	0/0.0	0/0.0	0/0.0	0/0.0	0/0.0	0/0.0	0/0.0	0/0.0	0/0.0	0/0.0	0/0.0
≥3(MDR)	76/100.0	7/100.0	9/100.0	92/100.0	152/92.7	37/77.1	64/91.4	16/100.0	11/64.7	280/88.9	372/91.4
≥5	75/98.7	6/85.7	9/100.0	90/97.8	109/66.5	34/70.8	46/65.7	15/93.8	11/64.7	215/68.3	305/74.9
≥7	65/85.5	5/71.4	5/55.6	75/81.5	50/30.5	16/33.3	9/12.9	4/25.0	5/29.4	84/26.7	159/39.1

Table S3 Fluoroquinolone and cephalosporins resistance genes in *S*. Indiana isolates from China, the United Kingdom, the United State and Germany.

Antibiotic resistance determination		Number of isolates (%)			
		China (n=171)	United Kingdom (n=322)	United State (n=82)	Germany (n=32)
GyrA	S83F/D87N	98/57.3[a]	2/0.6[b]	1/1.2[b]	0/0.0
	S83F/D87G	42/24.6[a]	2/0.6[b]	1/1.2[b]	0/0.0
	D87G	2/1.2	0/0.0	0/0.0	0/0.0
	S83F	3/1.8	2/0.6	0/0.0	0/0.0
	S83Y	0/0.0	6/1.9	0/0.0	0/0.0
	Total	145/84.8[a]	12/3.7[b]	2/2.4[b]	0/0.0
ParC	T57S/S80R	142/83.0[a]	4/1.2[b]	2/2.4[b]	0/0.0
	T57S	29/17.0[b]	318/98.8[a]	80/97.6[a]	32/100.0[a]
	Total	171/100.0	322/100.0	82/100.0	32/100.0
GyrA-ParC	S83F/D87N-T57S/S80R	98/57.3[a]	2/0.6[b]	1/1.2[b]	0/0.0
	S83F/D87G-T57S/S80R	42/24.6[a]	2/0.6[b]	1/1.2[b]	0/0.0
	S83F-T57S/S80R	1/0.6	0/0.0	0/0.0	0/0.0
	D87G-T57S	2/1.2	0/0.0	0/0.0	0/0.0
	S83F-T57S	2/1.2	2/0.6	0/0.0	0/0.0
	S83Y-T57S	0/0.0	6/1.9	0/0.0	0/0.0
	Total	145/84.8[a]	12/3.7 [b]	2/2.4[b]	0/0.0
aac(6')-Ib-cr		106/62.0[a]	4/1.2[b]	1/1.2[b]	0/0.0
*oqx*AB		51/29.8	0/0.0	0/0.0	0/0.0
*qnr*S2		29/7.6	5/1.6	0/0.0	0/0.0
*qnr*S1		0/0.0	1/0.3	0/0.0	0/0.0
*qnr*B1		0/0.0	2/0.6	0/0.0	0/0.0
*qep*A1		3/0.8	0/0.0	0/0.0	0/0.0
bla_{CTX-M}	$bla_{CTX-M-65}$	57/33.3[a]	2/0.6[b]	0/0.0	0/0.0
	$bla_{CTX-M-55}$	15/8.8	0/0.0	0/0.0	0/0.0
	$bla_{CTX-M-14}$	6/3.5[a]	0/0.0	1/1.2	0/0.0
	$bla_{CTX-M-27}$	6/3.5	0/0.0	0/0.0	0/0.0
	$bla_{CTX-M-15}$	1/0.6	0/0.0	0/0.0	0/0.0
	$bla_{CTX-M-123}$	3/1.8	0/0.0	0/0.0	0/0.0
	$bla_{CTX-M-90}$	1/0.6	0/0.0	0/0.0	0/0.0
	Total	89/52.0 [a]	2/0.6 [b]	1/1.2 [b]	0/0.0
bla_{CMY-2}		3/1.8	0/0.0	0/0.0	0/0.0
bla_{OXA-1}		106/62.0[a]	4/1.2[b]	1/1.2[b]	0/0.0
bla_{TEM-1B}		36/21.1[a]	8/2.5[b]	3/3.7[b]	2/6.3[b]

Different letters stand for significant difference (p<0.05)

Table S4 Primers used in this study for constructing mutations

Primer	Sequence (5′-3′)	Product size (bp)	Target
GyrA83-87F	ccccacggcgatttcgcagtgtataacaccatcgttcg		Introduced GyrA83 and/or 87 point mutations
GyrA83-87R	cgaacgatggtgttatacactgcgaaatcgccgtgggg		
ParC80F	gcttcatagcaggctctgtcgccatgcgg		Introduced ParC80 point mutation
ParC80R	ccgcatggcgacagagcctgctatgaagc		
GyrA-F	GCTCTAGACGGGATACAGTAGAGGGATAG	1093	*gyr*A
GyrA-R	CGAGCTCGCACGAACGCTGAAATGA		
ParC-F	GCTCTAGATGCGTTGCCGTTTATTGG	1154	*par*C
ParC-R	CGAGCTCCCTGGGTTTCGCTGATGC		
Pre112-F	GGTGCTGCCTCAGATTC		pRE112
Pre112-R	CCTGGCCTATTTCCCTA		

Table S5 Plasmids used in this study for constructing mutations

Plasmids	Description
pKOBEG	It is a helper plasmid with thermo-sensitive and apramycin resistance to increase the ability of homologous recombination through arabinose-inducible lambda red genes *gam*, *bet* and *exo*.
pMD™19-T	It is a special vector with ampicillin resistance for efficient cloning of PCR products (TA Cloning), which is derived from pUC19.
pRE112	It is a suicide plasmid with chloramphenicol resistance to make homologous recombination occurred through saccharose-inducible gene *sac*B.

Table S6 The MIC of ciprofloxacin in *S*. Indiana wild-type isolate and mutants

Isolates	*gyr*A83	*gyr*A87	*par*C80	*oqx*AB	MIC（μg/ ml）
SJTUF14139-WT	-	-	-		0.015
PML1	S83F	-	-		0.25
PML2	-	D87N	-		0.25
PML3	S83F	D87N	-		0.25
PML4	S83F	D87N	S80R		8
PML5	S83F	-	-	pMD*oqx*AB	1
PML6	-	D87N	-	pMD*oqx*AB	1
PML7	S83F	D87N	-	pMD*oqx*AB	2
PML8	S83F	D87N	S80R	pMD*oqx*AB	32

Table S7 Sequence data of *S*. Indiana in this study

Sample	Read_Num	Total_base	N_rate	GC_Content	Q20_rate	Q30_rate
SJTUF13786	7,877,968	1,181,695,200	0.0007	54.50	96.24	90.60
SJTUF13787	9,101,830	1,365,274,500	0.0007	54.30	96.59	91.31
SJTUF13788	8,145,970	1,221,895,500	0.0007	54.48	96.55	91.17
SJTUF13789	7,069,160	1,060,374,000	0.0007	52.86	96.66	91.49
SJTUF13791	6,733,794	1,010,069,100	0.0008	54.27	96.56	91.25
SJTUF13792	7,021,912	1,053,286,800	0.0007	54.53	96.68	91.50
SJTUF13794	8,635,948	1,295,392,200	0.001	54.16	96.60	91.39
SJTUF13803	6,927,456	1,039,118,400	0.0007	53.84	97.03	92.20
SJTUF13804	6,791,638	1,018,745,700	0.0007	53.95	96.70	91.56
SJTUF13810	6,810,738	1,021,610,700	0.0007	52.60	96.62	91.40
SJTUF13820	10,217,098	1,532,564,700	0.0009	53.35	97.25	92.27
SJTUF13829	7,208,652	1,081,297,800	0.0007	52.85	96.68	91.50
SJTUF13849	12,015,802	1,802,370,300	0.0009	52.91	97.39	92.51
SJTUF13851	7,259,014	1,088,852,100	0.0007	53.72	96.26	90.62
SJTUF13853	7,133,006	1,069,950,900	0.0007	53.51	97.10	92.42
SJTUF13873	7,518,698	1,127,804,700	0.0007	52.40	96.78	91.73
SJTUF13875	10,474,010	1,571,101,500	0.0009	53.55	97.61	93.11
SJTUF13876	14,644,570	2,196,685,500	0.0009	53.06	98.44	95.07
SJTUF13897	11,724,458	1,758,668,700	0.0009	53.06	97.33	92.45
SJTUF13900	10,860,106	1,629,015,900	0.0009	53.11	97.76	93.36
SJTUF13908	11,141,934	1,671,290,100	0.0009	53.10	97.58	92.97
SJTUF13911	8,603,850	1,290,577,500	0.0009	52.61	96.71	91.63
SJTUF13914	7,130,018	1,069,502,700	0.0007	52.86	96.89	91.89
SJTUF13929	8,864,094	1,329,614,100	0.0009	53.37	95.85	89.90
SJTUF13930	11,245,152	1,686,772,800	0.001	53.34	97.41	92.58
SJTUF13939	10,753,060	1,612,959,000	0.0009	52.79	97.17	92.04
SJTUF13944	10,447,492	1,567,123,800	0.0009	53.25	97.38	92.52
SJTUF13951	11,893,892	1,784,083,800	0.0009	53.00	97.39	92.52
SJTUF13954	8,207,780	1,231,167,000	0.0007	53.88	96.76	91.63
SJTUF13955	11,383,708	1,707,556,200	0.0009	53.40	97.54	92.88
SJTUF13957	12,183,278	1,827,491,700	0.0009	53.06	97.03	91.83
SJTUF13959	6,856,342	1,028,451,300	0.0007	54.07	96.97	92.09
SJTUF13960	12,115,870	1,817,380,500	0.001	52.92	97.13	92.06
SJTUF13980	10,512,722	1,576,908,300	0.001	53.10	97.30	92.44
SJTUF13981	7,065,940	1,059,891,000	0.0007	52.68	96.57	91.28
SJTUF13986	8,932,492	1,339,873,800	0.0009	52.85	96.86	91.88
SJTUF13987	7,328,204	1,099,230,600	0.0007	52.57	96.64	91.44
SJTUF13988	6,779,110	1,016,866,500	0.0007	52.96	96.88	91.91
SJTUF13990	8,732,928	1,309,939,200	0.001	52.66	97.93	93.86
SJTUF13999	7,104,788	1,065,718,200	0.0007	53.95	96.52	91.12
SJTUF14000	11,134,022	1,670,103,300	0.001	53.22	97.51	92.86
SJTUF14001	11,958,546	1,793,781,900	0.001	53.07	97.28	92.39
SJTUF14003	11,461,494	1,719,224,100	0.001	53.62	97.45	92.72
SJTUF14004	10,606,772	1,591,015,800	0.001	53.53	97.52	92.93
SJTUF14010	11,818,444	1,772,766,600	0.001	53.49	97.57	93.03
SJTUF14011	11,834,408	1,775,161,200	0.0009	53.36	97.49	92.79
SJTUF14013	11,777,714	1,766,657,100	0.0009	53.35	97.69	93.28
SJTUF14016	8,647,178	1,297,076,700	0.0003	52.69	97.11	92.10

SJTUF14017	7,336,838	1,100,525,700	0.0007	52.31	96.81	91.78
SJTUF14019	7,839,962	1,175,994,300	0.0003	52.71	97.27	92.38
SJTUF14020	7,652,748	1,147,912,200	0.0007	53.43	96.75	91.63
SJTUF14021	7,585,828	1,137,874,200	0.0004	52.46	97.05	91.87
SJTUF14023	8,476,520	1,271,478,000	0.0007	54.09	96.92	91.94
SJTUF14024	7,342,150	1,101,322,500	0.0007	54.38	96.98	92.13
SJTUF14025	6,767,536	1,015,130,400	0.0004	52.66	97.27	92.29
SJTUF14032	8,641,172	1,296,175,800	0.001	52.39	96.59	91.38
SJTUF14033	7,456,738	1,118,510,700	0.0004	52.75	97.09	91.85
SJTUF14034	7,581,478	1,137,221,700	0.0007	53.67	96.20	90.53
SJTUF14042	7,729,404	1,159,410,600	0.0004	52.59	97.58	92.90
SJTUF14043	6,901,224	1,035,183,600	0.0007	52.12	96.63	91.45
SJTUF14049	8,058,570	1,208,785,500	0.0008	52.98	97.06	92.30
SJTUF14051	14,235,646	2,135,346,900	0.0009	53.11	97.99	94.01
SJTUF14052	13,912,226	2,086,833,900	0.0009	53.21	98.24	94.53
SJTUF14053	14,838,318	2,225,747,700	0.0009	52.39	98.09	94.22
SJTUF14054	17,660,112	2,649,016,800	0.001	52.48	98.18	94.44
SJTUF14063	7,247,468	1,087,120,200	0.0008	53.58	97.06	92.35
SJTUF14065	13,092,926	1,963,938,900	0.001	53.06	97.25	92.24
SJTUF14071	8,367,350	1,255,102,500	0.0008	52.33	97.13	92.51
SJTUF14072	12,421,868	1,863,280,200	0.001	53.48	97.41	92.63
SJTUF14074	13,165,462	1,974,819,300	0.0009	52.30	98.32	94.79
SJTUF14075	12,834,820	1,925,223,000	0.0009	52.50	98.32	94.74
SJTUF14076	13,834,234	2,075,135,100	0.0009	52.85	98.19	94.52
SJTUF14077	9,718,290	1,457,743,500	0.001	53.49	97.22	92.24
SJTUF14078	13,363,824	2,004,573,600	0.0009	52.47	98.32	94.73
SJTUF14079	11,223,274	1,683,491,100	0.001	53.69	97.52	92.90
SJTUF14080	17,539,544	2,630,931,600	0.001	51.35	98.32	94.71
SJTUF14081	7,175,890	1,076,383,500	0.0007	52.45	96.69	91.50
SJTUF14082	14,035,356	2,105,303,400	0.0009	51.36	98.28	94.66
SJTUF14083	10,808,456	1,621,268,400	0.001	53.36	97.58	93.01
SJTUF14084	16,466,364	2,469,954,600	0.0009	51.43	98.25	94.58
SJTUF14087	16,736,536	2,510,480,400	0.001	52.91	98.33	94.85
SJTUF14088	10,717,024	1,607,553,600	0.001	53.51	97.72	93.34
SJTUF14090	7,659,628	1,148,944,200	0.0004	52.81	97.25	92.19
SJTUF14091	7,710,364	1,156,554,600	0.0004	52.87	97.31	92.29
SJTUF14092	7,589,306	1,138,395,900	0.0004	53.00	97.46	92.64
SJTUF14094	8,082,022	1,212,303,300	0.0004	52.71	97.24	92.17
SJTUF14095	7,586,864	1,138,029,600	0.0004	52.69	97.47	92.66
SJTUF14096	17,926,316	2,688,947,400	0.001	52.34	98.05	94.19
SJTUF14097	8,098,760	1,214,814,000	0.0004	52.18	97.85	93.53
SJTUF14098	16,073,062	2,410,959,300	0.001	52.29	98.23	94.54
SJTUF14099	17,872,380	2,680,857,000	0.001	52.73	98.08	94.25
SJTUF14100	15,626,434	2,343,965,100	0.001	52.68	97.99	94.03
SJTUF14101	17,202,204	2,580,330,600	0.001	52.10	98.34	94.87
SJTUF14102	14,532,996	2,179,949,400	0.001	51.35	98.35	94.84
SJTUF14103	15,654,762	2,348,214,300	0.001	52.36	98.13	94.36
SJTUF14104	14,948,632	2,242,294,800	0.001	52.41	98.30	94.72
SJTUF14105	17,293,918	2,594,087,700	0.001	52.81	98.20	94.53
SJTUF14106	15,070,252	2,260,537,800	0.001	52.95	98.26	94.68

SJTUF14107	14,466,764	2,170,014,600	0.0009	51.95	98.25	94.61
SJTUF14108	14,469,742	2,170,461,300	0.0009	51.97	98.35	94.85
SJTUF14109	15,843,528	2,376,529,200	0.0009	52.13	98.09	94.26
SJTUF14110	14,023,100	2,103,465,000	0.0009	52.40	98.32	94.75
SJTUF14111	16,328,444	2,449,266,600	0.001	52.54	98.21	94.49
SJTUF14112	15,710,494	2,356,574,100	0.0009	52.24	98.28	94.78
SJTUF14113	15,296,302	2,294,445,300	0.0009	52.51	98.33	94.84
SJTUF14114	16,885,952	2,532,892,800	0.001	52.30	98.36	94.84
SJTUF14115	13,394,242	2,009,136,300	0.0009	53.69	98.18	94.51
SJTUF14116	13,117,198	1,967,579,700	0.0009	52.77	98.34	94.79
SJTUF14117	16,674,824	2,501,223,600	0.0009	52.93	98.20	94.52
SJTUF14119	11,508,514	1,726,277,100	0.001	53.27	97.54	93.01
SJTUF14123	9,948,412	1,492,261,800	0.0009	53.09	97.16	92.09
SJTUF14124	9,872,926	1,480,938,900	0.0009	53.53	97.48	92.86
SJTUF14132	11,032,990	1,654,948,500	0.001	53.48	97.60	93.06
SJTUF14137	10,915,534	1,637,330,100	0.001	53.16	97.72	93.34
SJTUF14138	8,565,290	1,284,793,500	0.0007	53.50	96.42	90.96
SJTUF14139	11,239,384	1,685,907,600	0.001	53.24	97.41	92.66
SJTUF14142	10,644,666	1,596,699,900	0.001	52.67	96.95	91.66
SJTUF14144	11,688,528	1,753,279,200	0.001	53.05	97.27	92.35
SJTUF14146	11,272,972	1,690,945,800	0.001	53.03	97.85	93.71
SJTUF14149	7,307,864	1,096,179,600	0.0007	53.45	96.09	90.32
SJTUF14150	9,555,178	1,433,276,700	0.0009	53.33	97.26	92.37
SJTUF14151	14,447,722	2,167,158,300	0.001	52.34	98.06	94.16
SJTUF14152	13,708,758	2,056,313,700	0.001	54.06	98.27	94.71
SJTUF14153	7,688,188	1,153,228,200	0.0007	53.93	96.87	91.90
SJTUF14154	11,222,796	1,683,419,400	0.0009	53.27	97.45	92.74
SJTUF14156	14,954,778	2,243,216,700	0.0009	52.73	98.29	94.72
SJTUF14158	14,347,964	2,152,194,600	0.0009	53.76	98.07	94.26
SJTUF14161	15,298,914	2,294,837,100	0.0009	53.76	98.00	94.06
SJTUF14162	16,199,986	2,429,997,900	0.0009	52.60	98.45	95.16
SJTUF14166	13,585,208	2,037,781,200	0.0009	52.95	98.3	94.72
SJTUF14167	14,658,516	2,198,777,400	0.0009	52.65	98.15	94.45
SJTUF14169	13,848,304	2,077,245,600	0.0009	53.80	98.24	94.60
SJTUF14170	16,240,864	2,436,129,600	0.0009	53.22	98.17	94.44
SJTUF14171	14,211,556	2,131,733,400	0.0009	53.02	98.27	94.69
SJTUF14172	15,286,860	2,293,029,000	0.001	53.21	98.27	94.71
SJTUF14173	10,753,918	1,613,087,700	0.001	53.71	97.24	92.35
SJTUF14176	7,619,988	1,142,998,200	0.0008	53.40	96.98	92.10
SJTUF14178	15,022,932	2,253,439,800	0.001	52.73	98.07	94.24
SJTUF14179	14,966,564	2,244,984,600	0.001	52.78	98.28	94.67
SJTUF14180	16,463,882	2,469,582,300	0.0009	53.36	98.14	94.43
SJTUF14183	15,626,746	2,344,011,900	0.001	53.25	98.01	94.09
SJTUF14184	10,108,866	1,516,329,900	0.001	53.58	97.40	92.64
SJTUF14185	7,089,802	1,063,470,300	0.0008	53.47	97.25	92.73
SJTUF14187	11,136,912	1,670,536,800	0.001	53.14	98.32	94.78
SJTUF14188	12,686,564	1,902,984,600	0.001	53.28	98.17	94.48
SJTUF14190	12,666,988	1,900,048,200	0.001	54.02	98.35	94.89
SJTUF14191	15,141,414	2,271,212,100	0.001	52.93	98.23	94.61
SJTUF14192	15,937,286	2,390,592,900	0.001	52.15	97.93	93.89

SJTUF14193	12,712,454	1,906,868,100	0.001	54.13	98.31	94.82
SJTUF14194	12,859,210	1,928,881,500	0.001	53.90	98.36	94.92
SJTUF14195	7,950,666	1,192,599,900	0.0009	52.63	96.83	91.89
SJTUF14197	12,332,728	1,849,909,200	0.001	53.68	98.33	94.78
SJTUF14198	15,239,386	2,285,907,900	0.001	52.34	98.12	94.32
SJTUF14200	11,056,634	1,658,495,100	0.001	52.87	97.39	92.59
SJTUF14201	12,992,934	1,948,940,100	0.001	53.63	98.06	94.20
SJTUF14202	12,898,838	1,934,825,700	0.001	53.93	98.36	94.94
SJTUF14203	12,715,062	1,907,259,300	0.001	53.94	98.29	94.70
SJTUF14204	12,584,370	1,887,655,500	0.001	53.11	98.19	94.51
SJTUF14205	11,971,406	1,795,710,900	0.001	53.16	98.34	94.83
SJTUF14212	10,799,864	1,619,979,600	0.001	53.43	97.49	92.84
SJTUF14213	13,638,588	2,045,788,200	0.001	53.13	98.22	94.59
SJTUF14216	11,554,074	1,733,111,100	0.001	54.11	98.34	94.89
SJTUF14217	13,286,176	1,992,926,400	0.001	53.97	98.24	94.65
SJTUF14218	10,147,708	1,522,156,200	0.001	53.22	97.73	93.39
SJTUF14219	16,599,296	2,489,894,400	0.001	53.20	98.30	94.75
SJTUF14220	15,905,886	2,385,882,900	0.001	53.86	98.04	94.25
SJTUF14221	13,118,982	1,967,847,300	0.001	52.77	98.26	94.59
SJTUF14222	15,130,116	2,269,517,400	0.001	53.28	98.13	94.36
SJTUF14227	14,579,848	2,186,977,200	0.001	52.48	98.00	94.05
SJTUF14284	11,286,640	1,692,996,000	0.001	52.93	97.06	91.85

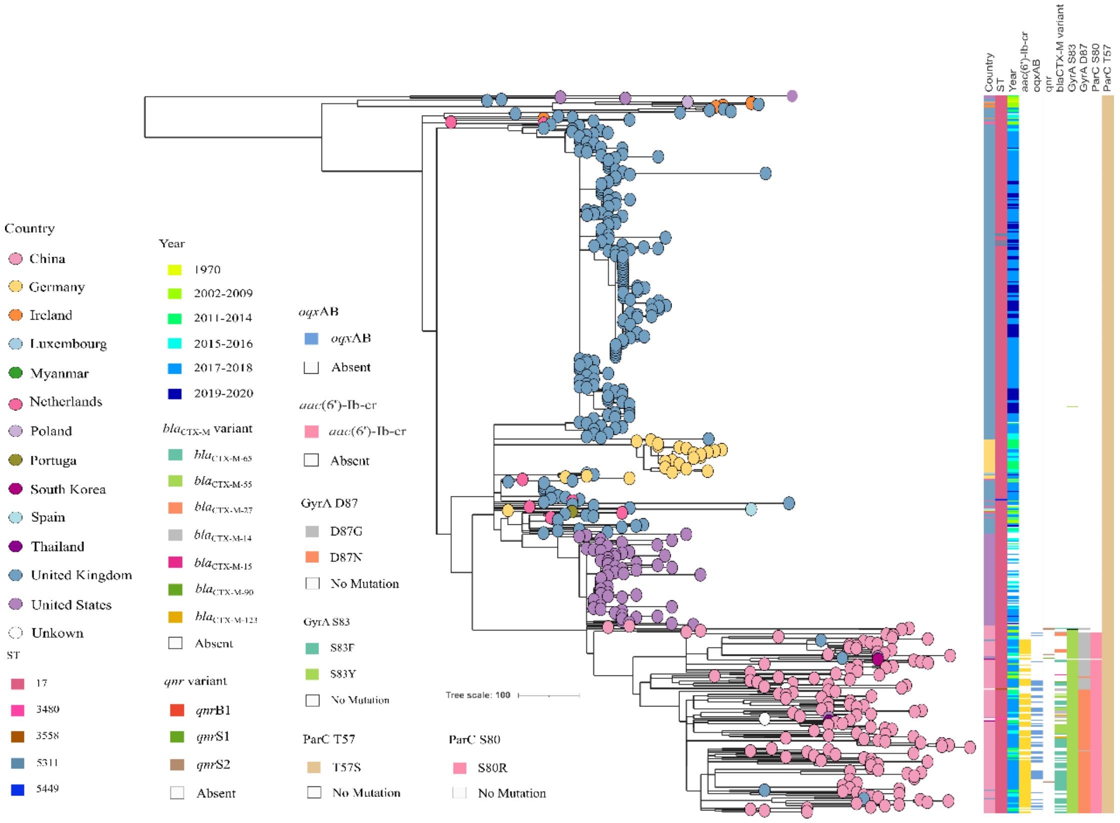

Fig. S1 Phylogenetic tree of ST17 *S*. Indiana isolates, extracted from the tree in Fig.2**.** Leaf nodes are colored by country (see the key). The colours of the isolates tips represent years of isolation and metadata columns including *bla*$_{\text{CTX-M}}$ variants, PMQR genes (*oqxAB*, *qnr* and *acc(6')-Ib-cr*) and QRDR mutation (GyrA S83/D87 and ParC T57/S80).

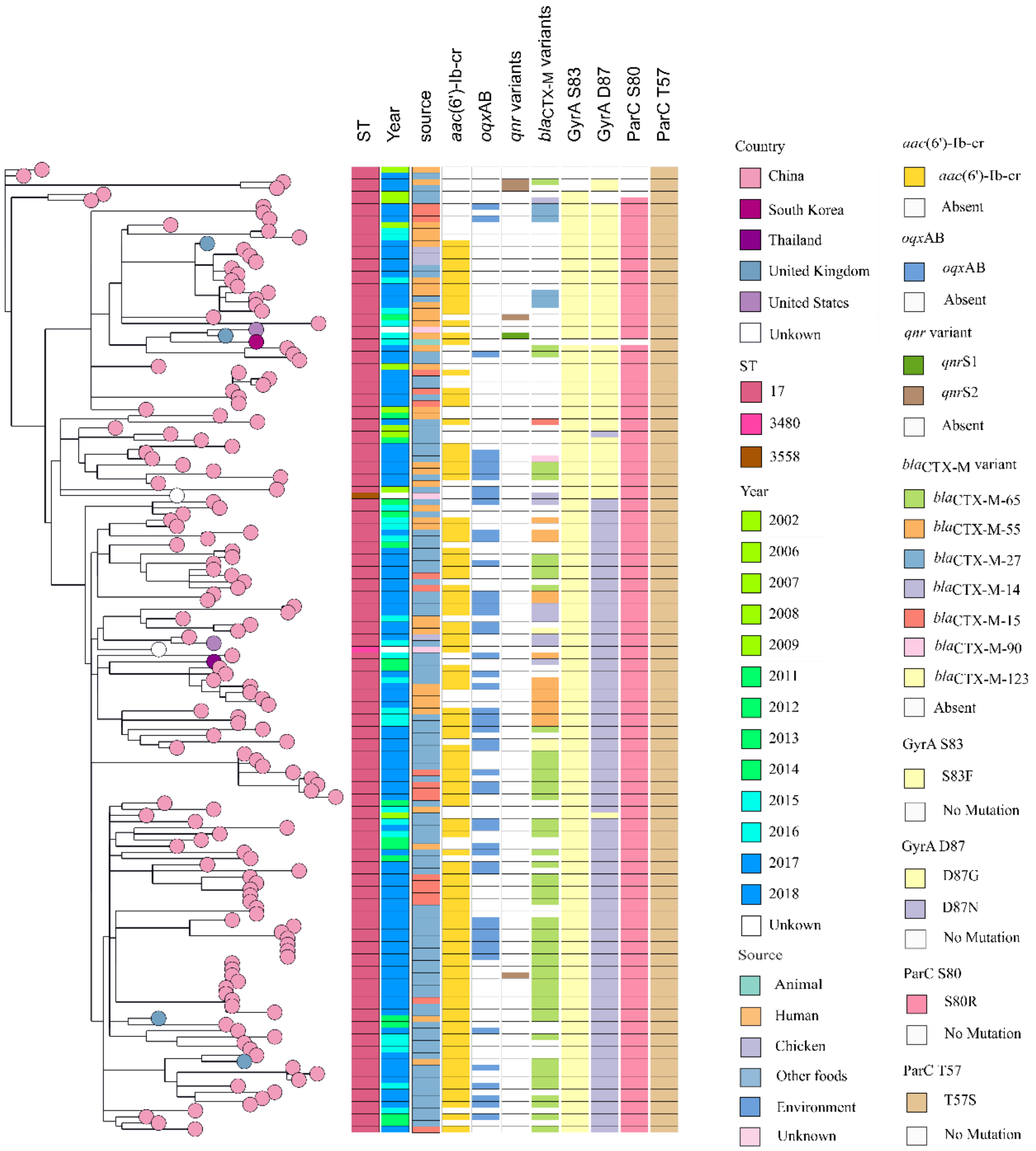

Fig. S2 Phylogenetic subtree of clade IV isolates, extracted from the tree in Fig.2**.** Leaf nodes are colored by country (as shown in the inset legend). The colors of the isolates tips represent metadata columns including MLST types, years, sources, *bla*$_{CTX\text{-}M}$ variants, PMQR genes (*oqx*AB, *qnr* and *aac*(6')-Ib-cr) and QRDR mutation (GyrA S83/D87 and ParC T57/S80) (as shown in the inset lenged). *bla*$_{CTX\text{-}M}$ variants columns included *bla*$_{CTX\text{-}M\text{-}65}$, *bla*$_{CTX\text{-}M\text{-}55}$, *bla*$_{CTX\text{-}M\text{-}14}$, *bla*$_{CTX\text{-}M\text{-}27}$, *bla*$_{CTX\text{-}M\text{-}123}$, *bla*$_{CTX\text{-}M\text{-}15}$ and *bla*$_{CTX\text{-}M\text{-}90}$ (as shown in the inset legend).

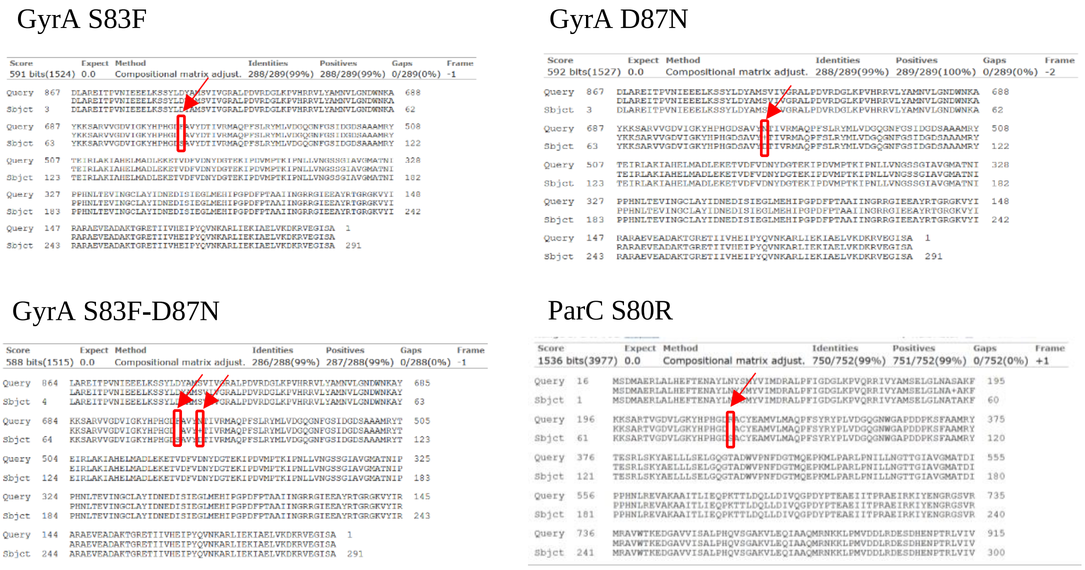

Fig. S3 Sequence and BLASTP results of constructed isolates of GyrAS83F, GyrAD87N, GyrAS83F-D87N and GyrAS83F/D87N-ParCS80R

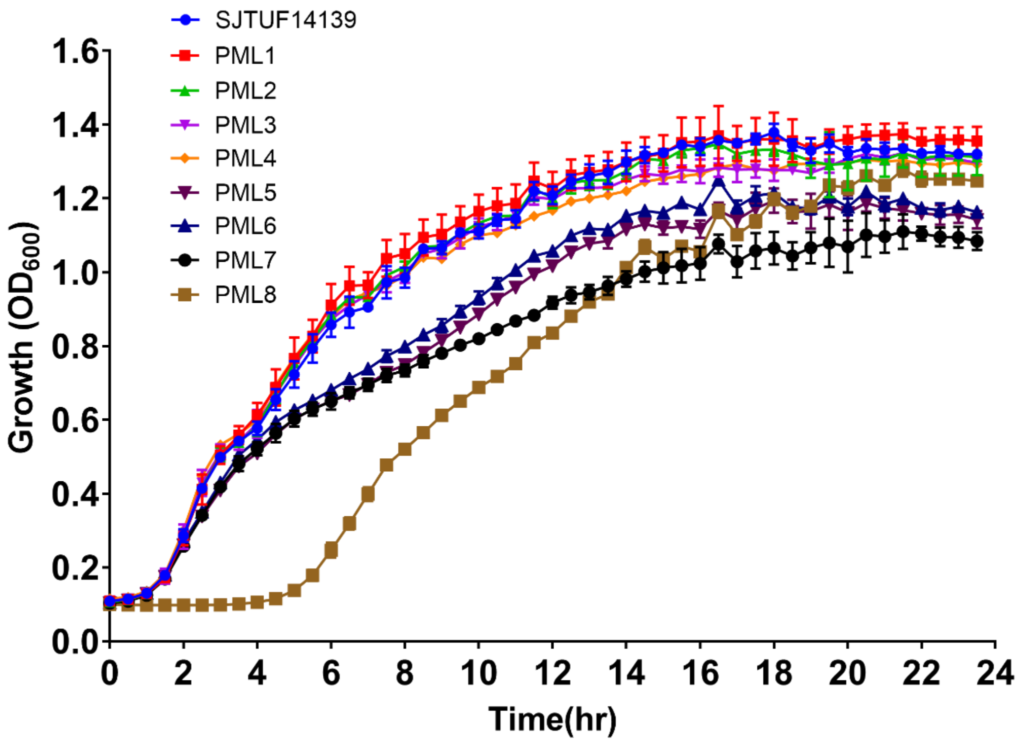

Fig. S4 Growth curves of wild-type *Salmonella* Indiana SJTUF14139 and its site-directed mutants in LB broth. PML1 (*gyr*AS83F), PML2 (*gyr*AD87N), PML3 (*gyr*AS83F/D87N), PML4 (*gyr*AS83F/D87N-*par*CS80R), PML5 (*gyr*AS83F-pMD*oqx*AB), PML6 (*gyr*AD87N-pMD*oqx*AB), PML7 (*gyr*AS83F/D87N-pMD*oqx*AB) and PML8 (*gyr*AS83F/D87N-*par*CS80R-pMD*oqx*AB).
